# Breaking Up Rationally

**DOI:** 10.1007/s10892-024-09505-5

**Published:** 2025-01-15

**Authors:** Daniel Villiger, Bouke de Vries

**Affiliations:** 1 Institute of Philosophy, University of Zurich, Zollikerstrasse 117, 8008 Zurich, Switzerland; 2 Uehiro Oxford Institute, University of Oxford, Oxford, UK; 3 Department of Philosophy and Moral Sciences, Ghent University, Ghent, Belgium

**Keywords:** Romantic relationships, Breakups, Rational choice theory, Transformative experiences, Rationality

## Abstract

The end of a long-term romantic relationship ranks among the most stressful and momentous events in life. Thus, the decision of whether to break up with someone whom one has been with for many years should generally be made very carefully. Unfortunately, decision theory is often thought to be unable to provide rational guidance in such high-stake life choices due to the outcomes’ presumed transformative character. The present paper shows how agents can rationally decide whether to leave their romantic partner *even if* the decision is transformative. It does so by using a novel five-level account of transformative decision-making, which can also be used for other key life choices, and which is the first to integrate in a systematic way several approaches for making (certain types of) transformative decisions that have been proposed in recent years.

## Introduction

A fulfilling romantic relationship can be one of the most valuable goods in life. In such relationships, we can find love, care, intimacy, appreciation, company, security, and much more (Ge et al. 2020; Gheaus 2018), which helps to explain why the majority of people want to be in one (Brown 2020).^1^ The flipside of this is that breaking up with one’s romantic partner can be, and frequently is, difficult to bear. In a ranking of most stressful life events, divorce is on the 7th place and non-marital separation on the 12th place, making them more stressful than for example the death of a close friend or the survival of a natural disaster (Hobson et al. 2001). While particularly stressful for a unilaterally left partner,^2^ studies have found that breakups can take a heavy toll on unilaterally leaving partners and bilaterally leaving partners alike (Locker et al. 2010; Sprecher et al. 1998, Steiner et al. 2011; 2015). In addition to this, it has been reported that even years after a divorce or non-marital separation, these events are associated with lower levels of life satisfaction (Amato and Hohmann-Marriott 2007; Gustavson et al. 2012; Leopold and Kalmijn 2016).^3^

Given these findings, the decision to end a relationship should not be made imprudently (cf. Betzler 2023). One prominent theory that can be helpful in this regard is rational decision theory, which leads to the following question: When is it rational to end, or not to end, a relationship? Although breakups are a widespread phenomenon—most estimates say that there is a 30% to 50% likelihood that a marriage in the US will end in divorce (Liberman 2021), with the breakup rate for unmarried couples being much higher still (Rosenfeld 2014)—this specific question has not been addressed from the perspective of this theory.

Some philosophers have worked on whether divorce involves promise-breaking (e.g., Brake 2011; Cowley 2020; Liberman 2021), which is an important related issue but one we set aside here, given that many relationships are non-marital. Others have considered when, if ever, loving someone can be rational, a-rational, or irrational respectively (e.g., Brogaard 2015; Jollimore 2017; Kolodny 2003; McKeever and Saunders 2022; Naar 2022). Despite carrying certain insights for our research question—especially the paper by Kolodney (2003) given that he locates the reasons to love someone in the reasons to value the shared relationship (more on this later)—this issue is relevantly separate from it, as loving someone and being in a relationship with them are not the same thing; we can love individuals with whom we do not share a romantic relationship, as well as be in a romantic relationship with someone we no longer love if we ever did (cf. Saunders 2022: 136). Accordingly, even if loving a given person P can be, and is, rational in certain cases, it does not follow that it must be rational to start or maintain a romantic relationship with P (perhaps because being in a relationship with us would, or does, make P unhappy, or perhaps because it would, or does, impose high costs on third parties), just as the fact that loving another individual P* may be irrational or a-rational does not imply that it must be irrational or a-rational to have a romantic relationship with that person (perhaps there are good financial reasons to have a (continued) relationship with P*, or perhaps breaking up with them would be highly detrimental to any shared children). Still other related philosophical work has respectively sought to:

- offer a reason-based account of what it is for love to pass (Saunders 2022);
- phenomenologically capture the potentially disorienting experience of falling out of love (Cowley 2021);
- explore the potential prudential and moral value of falling out of love (Lopez-Cantero and Archer 2020);
- explicate the object of grief when we break up (Lopez-Cantero 2018);
- consider the distinct moral rights and duties of those who unilaterally leave their partner (Betzler 2023).

While we will see in due course that this body of research also offers certain relevant insights for this paper, what is important for present purposes is that none of it looks in depth at the rationality, or lack thereof, of breakups.

At the same time, since Paul’s (2014) groundbreaking book *Transformative Experience,* the question of whether we can make life choices rationally has received much attention in analytic philosophy. In brief, Paul argues that the transformative nature of life choices impedes decision theory by obscuring which option has the highest expected value. Interestingly, while partnership choices, such as having children or getting married, are among the most prominent examples in the literature on transformative experiences, the termination of romantic relationships remains underexplored (e.g., Barnes 2015; Paul 2014; 2015c; Reuter and Messerli 2018; Villiger 2021).

The present paper helps to close this gap by showing how agents can rationally decide whether to leave their romantic partner in the most difficult cases, namely ones where a breakup is transformative. In doing so, the paper focuses primarily on prudential values, as these are the ones that the transformative nature of an outcome is supposed to obscure. We begin by arguing that breakups frequently have a transformative character and explain how this complicates the decision to end a romantic relationship (Sect. 2). Next, we demonstrate how rational decisions about transformative breakups can be reached nonetheless using a novel five-level account of transformative decision-making, where one advances to the next level only if the previous one did not enable rational choice. First, one considers whether moral values rationally determine what to do, which we suggest they might in certain breakup decisions (Sect. 3). Second, one tries to restore rationality by running an approximate mental simulation of breaking up based on so-called “higher-order facts”, which are facts learned from previous experiences that also apply to the outcome of breaking up (Sect. 4). At this, we suggest that for instance higher-order facts learned from previous breakups, past times of singledom, and/or losses of loved ones can enable approximate mental simulations of a (next) breakup. Third, one considers whether one can directly base one’s decision on the values derived by those who have already undergone the transformative experience (Sect. 5). At best, we attain those values from well-matched testimony. If this is not possible, we can also attain it from empirical studies, provided that their results are fairly clear-cut. Fourth, one tries to cut down the decision into sub-decisions of which at least the first is non-transformative and at best all are non-transformative (Sect. 6). As we argue, one way to cut down the decision whether to separate is to decide first whether to have a relationship break, and after that break, whether to separate. Fifth, one bases the decision of whether to break up on the revelatory value, that is, one does (not) break up if one does (not) want to find out what it is like to no longer be in the relationship (Sect. 7). The final section concludes (Sect. 8).

Before delving into these matters, we should highlight that this five-level account can also be used for other transformative decisions, including ones about whether to have children; whether to make life-altering career shifts; and whether to relocate to a culturally radically different part of the world.^4^ As such, its practical significance is not limited to decisions about romantic breakups. Another thing to note about the five-level account is that, to the best of our knowledge, it is the first to highlight the importance of moral values in transformative decision-making. Also, it is the first to integrate in a systematic way several plausible approaches for making (certain types of) transformative decisions that have been proposed in recent years, such as those by Ullmann-Margalit (2006); Dougherty et al. (2015); Pettigrew (2015, 2016a, b, 2019); Sharadin (2015); and Paul (2014). That is, rather than being rival decision-making procedures, this paper shows that many of these approaches can, and should, be part of a more comprehensive strategy for making transformative decisions. Accordingly, this paper contributes not only to the literature on separation, but also to the literature on transformative experiences more generally.

## The Challenge of Transformative Experiences and How Breakup Decisions Are Affected by It

Paul’s (2014) book *Transformative Experience* has triggered an extensive debate on the question of whether life choices can be made rationally. Paul herself is rather pessimistic in this regard. In her view, the normative standard for rational decision-making is that the agent should choose the option which maximizes (expected) value. Rational decision theory provides the procedure how to determine which of the available option that is. First, the agent considers one of the available options and the potential outcomes that option involves. Second, the agent assigns the value of each of the option’s potential outcomes. Third, the agent multiplies the value of each potential outcome with the outcome’s probability. Fourth, the agent adds up all these products and thereby attains the option’s expected value. Fifth, the agent does so with every available option, ultimately telling them which option has the highest expected value.

The presence of transformative experiences poses a twofold challenge to the second step of this rational decision-making procedure. First, we cannot anticipate an outcome’s value if we have not experienced that kind of outcome before because only by experiencing the outcome, we get to know its value. So, experiencing the outcome involves an *epistemic transformation* which blocks the outcome’s value before experiencing it (in the next section, we will see that the epistemic transformation only obscures the subjective value of the outcome, but this is already sufficient to make the total value of the outcome unevaluable). Second, experiencing the outcome can also lead to a *personal transformation*, which means that it changes our very preferences. The possibility of a personal transformation further complicates rational decision-making as it becomes unclear on which preferences the decision should be based and what these preferences are.^5^

The literature’s most prominent example of a transformative experience is becoming a parent (e.g., Barnes 2015; Cath 2019; Kind 2020; Paul 2014, 2015c; Pettigrew 2015, 2019; Reuter and Messerli 2018; Villiger 2021, 2022). On the one hand, only by becoming a parent you know how it is to be a parent. On the other hand, becoming a parent can change your preferences: outcomes you have highly valued as a non-parent as for example going out with friends are no longer valued that much, whereas other outcomes such as spending time at home have gained value. Both the epistemic and the potential personal transformation make the (expected) value of becoming a parent inaccessible. Because of this, you cannot compare the expected value of becoming a parent with that of not becoming a parent, leaving you ignorant of which option maximizes expected value. Rational decision-making runs into a dead end.

Is the challenge that transformative experiences pose to decision theory also relevant when deciding whether to leave one’s partner? Since not all breakups seem to be transformative, we believe that the answer must be “not always”. Consider, for instance, a case where following conditions are satisfied:

i. you have recently started a new romantic relationship, say four months ago;
ii. you have ended several early-stage relationships over the past decade;
iii. you have recent knowledge of what being single is like—perhaps you lived as one for a year before starting your current relationship.

Under these conditions, the fact that you have recently been single (making it unlikely that you will experience singleness much differently than you did then) and are familiar with ending early-stage relationships suggests that you are likely able to predict the value of ending your current relationship. At the same time, the fact that continuing this relationship preserves the status quo means that there is a high probability that you can anticipate the value of the other option as well, namely that of staying in the relationship. Taken together, then, these considerations suggest that there are unlikely to be problems for rational decision theory.

But what about cases where the relationship has been more long-lasting (e.g., ten years) and where the lives of the partners are closely intertwined (e.g., you share an apartment and possibly even have children together)? In such cases, it strikes us that the experience of leaving your partner will often be transformative in one or two ways: First, it will be epistemically transformative if it causes you to experience an unfamiliar outcome and thereby get to know its value. This is clearest when you go through the breakup of your first serious relationship. However, even if you have experienced the termination of a serious relationship before, later breakups may be very different from earlier ones because the person you are leaving and the relationship you had with them will be different. And even if you do break up with the same person again (as in the case of Jennifer Lopez and Ben Affleck, for example), it can still be transformative. This is because non-relationship factors such as your personal circumstances or you as a person may have changed significantly since your first breakup. Besides, the fact that the relationship did not work out *again* may have a major impact on the experience of ending it.

Second, it will be personally transformative if the experience changes your preferences about how to fashion your life and possibly even your fundamental self-understanding (What kind of person am I? What do I value or stand for?). To appreciate how this works, it should be noted that when you have built your life, or simply a large part of it, around your relationship (e.g., you have children with your partner, you share many friends, you have done most recreational activities together over the past years), various areas of your life might begin to unravel after a breakup, necessitating an extensive re-building. Given that, as various authors have observed (Cowley 2020, 2021; Lopez-Cantero and Archer 2020), both what we value and how we conceive of ourselves at a basic level tends to be significantly shaped by those we share a committed romantic relationship with, this need to rebuild one’s life can, and often does, lead people to rethink and ultimately change their priorities and self-understanding.^6^ (Note that such a personal transformation will also be *epistemically transformative*, assuming that you cannot anticipate whether and how the breakup will personally transform you.)

When breakups are transformative in one or both of these ways, (traditional) decision theory cannot tell us whether ending the relationship has higher expected value than staying in it. While problematic in its own right, this is especially unfortunate since the stakes of the decision to part ways tend to be highest in such cases—just contrast a case where, all else being equal, you leave your partner after three months with one where you leave your partner after having spent six years together. Thus, in those situations where the potential consequences of choice are most severe, decision theory seems unable to provide us with guidance.

At this point, one might object that decision theory is misplaced in the context of breakups anyway, so its presumed inability to provide guidance in this area is of little concern: Staying with or leaving someone is often something people just do without thinking rationally about it, with many experiencing it as a necessity rather than a choice. Moreover, if you begin to think about your relationship in decision-theoretic terms, you may already be undermining what it means to truly be in a relationship.

While these concerns are important, we do not think that they make a decision-theoretic analysis of breaking up superfluous. First, as mentioned before, the literature’s most prominent example of a transformative decision is whether to become a parent. Like breaking up, starting a family is something people often just do without thinking rationally about it. However, this does not imply that you should not consider this decision from a rational perspective, as the extensive literature on the subject demonstrates. Second, relationship ambivalence is a common phenomenon that can result in uncertainty about whether to maintain a relationship (Trachsel et al. 2012; Zayas, Surenkok, and Pandey 2017; Zoppolat et al. 2024). So even though for some people staying with or leaving someone is not experienced as a choice, for others it is, and that choice can be agonizing (this is probably similar to the choice of becoming a parent). Third, while it may seem odd or even wrong to think about a relationship in terms of rationality, it ultimately comes down to asking which option (i.e., staying or leaving) you have the most reason to choose (as we will see in the next section, this does not exclude other-regarding considerations). Asking this question, for example, in the presence of relationship ambivalence, seems entirely appropriate to us: you should only leave your partner if you have more reason to do so than to stay with them (and vice versa). Therefore, we do not believe it is misguided to think about relationships in decision-theoretic terms (any more than it is to think about parenthood in such terms). The problem is that the transformative nature of breaking up seems to obscure how much reason we have to choose this option. This brings us back to the question: Is there a rational way to decide about a transformative breakup?

## Consider Moral Values (Level I)

We think there is. To be more precise, we believe that there exists a five-level account of making such (and other transformative) decisions rationally, whereby one proceeds to the next level only if the previous one does not allow one to reach a rational verdict. According to this strategy, which we will detail in the current section and the subsequent four, one starts by considering whether moral values rationally determine whether to end the relationship or not. If they do, the challenge that the transformative character of the outcome poses to decision theory becomes irrelevant. This is why considering moral values is the first level in our five-level account of transformative decision-making.

If we analyze a transformative outcome’s value more closely, we realize that only part of it is blocked, namely the outcome’s subjective value. The subjective value is experientially grounded and comprises the assessment of the nature of what it is like to *live* an outcome (Paul 2015a). According to Paul, an agent assesses an outcome’s subjective value by running a mental simulation of what it would be like if the outcome were to occur. But running such a mental simulation is possible *only if* the agent has experienced that kind of outcome before, at least if the mental simulation is to have some degree of reliability. What this means is that the agent cannot mentally simulate a transformative outcome and, consequently, not assess the transformative outcome’s subjective value nor its overall value of which its subjective value is a part.

By contrast, an outcome’s non-subjective values refer to its non-experiential aspects and thus do not need mental simulation for their assessment. Reuter and Messerli (2018) and Kauppinen (2015) have proposed accounts of rational transformative decision-making that build on non-subjective values. On the former’s view, non-subjective values can sometimes be sufficient to reach a rational decision even if it is transformative such as when deciding whether to have a child. This is because criteria other than how it is to live the outcome of being a parent (e.g., consistency with previous goals, outcome of discussion with partner, financial costs, etc.) can be of such combined relevance that, together, their values make the subjective-value-criterium irrelevant for the decision. Applied to the decision of whether ending or staying in a relationship, someone could for example rationally stay in a relationship if the economic costs of leaving the partner are sufficiently high to make the subjective-value-criterium irrelevant.^7^ In contrast, the account of Kauppinen (2015) states that life choices should generally be made from a story-regarding perspective and not an experience-regarding perspective. In other words, we should principally base our life choices on whether they build on past efforts and experiences and are consistent with our commitments and not on how it is to experience the chosen outcome. For example, someone who feels restricted in their relationship can rationally end it if they have a self-narrative of being free and independent.

Paul (2015a) rejects these non-subjective value accounts of rational transformative decision-making. She argues that the mere knowledge of non-subjective values is hardly ever sufficient to determine the right choice in the choices she is interested in (she calls them “first-personal choices”). This is because an outcome’s unconsidered subjective value might be so positive or so negative that it swamps non-subjective values, making them no longer decisive. And ignoring the subjective value altogether as Kauppinen does is indefensible in her view. Paul (2014) writes: “If, as a member of an affluent, contemporary Western culture, you dispense with subjective deliberation and subjective values in today’s world, you reject a central tenet of that culture’s ordinary way of thinking about the choice.” (85).

Paul’s arguments against the accounts of Reuter and Messerli (2018) and Kauppinen (2015) strike us as conclusive (cf. Villiger 2023). However, we think that these accounts have ignored one type of non-subjective value that might make rational transformative decisions possible in certain cases, namely moral values. Paul (2014: 25) acknowledges that moral values bear on rational decision-making and may sometimes even be decisive, just not in the case of first-personal choices. While this may be plausible for the transformative decision of whether to have a child, it is much less so for the decision of whether to leave your partner, given the potential of profoundly negative effects for both your partner and for third parties (see the introduction and our discussion below).^8^

For the purposes of this article, we will not try to settle whether moral values are ever decisive in deciding about a transformative breakup. Doing so would require us to do three things:

I. Defend a view of rationality on which the rational course of action is not wholly determined by what is in our self-interest (as prudence-centered accounts of rationality maintain), but rather by what we have all-things-considered reason to do.
II. Show that moral reasons can, and sometimes do, trump reasons stemming from other sources of normativity, including prudential normativity,^9^ as various authors believe (e.g., for Gauthier (1986), Kant (1996 [1797]), and Korsgaard (2009), performing the morally optimal action is *always* rational; for skepticism and possible counterexamples, see Copp (2015)).
III. Show that condition (II) applies to at least some instances where people are deciding about a transformative breakup.

Since tasks (I) and (II) are well beyond this article’s scope, we confine ourselves here to suggesting that *if* both tasks can be achieved, then the notion that people should sometimes opt for a transformative breakup on moral grounds (III) becomes highly probable. To bring this out, it bears noting that some of us have strong moral reasons to terminate our romantic relationship *even when* the outcome is likely to be transformative. This is clearest when a breakup is necessary for people to ensure that their minor children receive a minimally decent upbringing (Austin 2016; Blustein 1982; Brighouse and Swift 2014; O’Neill 1979). Examples of this might include, but are not necessarily limited to:

- Cases where you have minor children who are being abused physically and/or emotionally by your partner and where ending the relationship is the only way to stop, or simply reduce the incidence of the abuse^10^;
- Cases where your partner is “only” abusive towards you and where the mental and/or physical harm this causes is significantly compromising your ability to look after your minor children (Chiesa et al. 2018);
- Cases where your minor children regularly witness such abuse, which has been associated with a variety of behavioral, emotional, and cognitive-functioning problems (Edleson 1999; Johnsona et al. 2002);
- Cases where there is much conflict between you and your partner (but not necessarily abuse) that is unlikely to be resolved, as there is evidence that “growing up in a conflict-ridden but stable family can have more negative effects on children’s psychological well-being than parental separation” (Härkönen et al. 2017: 166-67).

Besides such child-centric reasons for terminating one’s romantic relationship, some of us have strong moral reasons to break up based on *our partner’s interests*. This will be true, for instance, when we no longer feel any affection for our partner and we know that they only want to be in a relationship with someone who has affection for them. Finally, we should mention the possibility that some of us possess robust *self-regarding moral reasons* to end our romantic relationships. Such cases may exist, for instance, when we are treated poorly by our partner (perhaps they are being sexually, emotionally, and/or financially unfaithful; cf. Blow and Hartnett, 2005; Buss, 2018; Garbinsky et al., 2020), and possibly also when we simply no longer derive any satisfaction or meaning from sharing our life with them (cf. Saunders 2022: 135). However, since the notion that we have (strong) self-regarding moral reasons is more controversial than our possession of strong other-regarding moral reasons (for an overview of the literature, see Muñoz, 2022), we do not want to put too much weight on this kind of possible justification here. What matters for the purposes of this article is simply that, as the child-centric cases discussed most clearly show, there are romantic partners with compelling moral reasons to end their relationship *even when* the outcome is likely to be transformative. If correct, then to the extent that the rationality of our actions is shaped significantly by our moral reasons (see above), this would suggest that opting for a transformative breakup on moral grounds can at least sometimes be expected to be rational.^11^

Before moving on, we should note that in a widely discussed paper on justifications for love, Niko Kolodny (2003), has proposed a different kind of non-subjective value that could potentially determine the (ir)rationality of ending a romantic relationship. In his view, having a romantic relationship where you and your partner show levels of concern for each other that, on the whole, are not outweighed by antagonistic feelings, such as anger or resentment, is itself a decisive reason to continue the relationship, as well as one to continue loving the other person, which is the paper’s focal point.^12^ As he explains, breaking up under these conditions would be inappropriate, yet not necessarily constitute a moral or prudential failure (Kolodny 2003: 163). What is pertinent for us is that *if* he is right about this, then this widens the range of non-subjective values that might render it irrational to opt for a transformative breakup. However, since this view is controversial—many philosophers have questioned whether our relationships as such (i.e., non-derivatively) can provide us with practical reasons, and ones to love our partner specifically (for an overview, see Helm 2021)—we do not wish to put too much weight on this possibility here.

## Simulate the Outcome Based on Higher-Order Facts (Level II)

What if moral values do not settle the question of whether it is rational to break up or not—suppose, for instance, that despite being a non-abusive, caring, and faithful person, your partner simply no longer adds much happiness to your life? We propose the following strategy for handling such cases: simulate the outcome based on higher-order facts. The method of using higher-order facts—meaning facts about the higher-order properties of an experience—for rational transformative decision-making was introduced by Paul (2014: 37-38). When discussing the experience of eating durian—an exotic fruit with a unique taste—Paul argues that we can approximately assess the range of possible subjective values. This is because we have eaten different kinds of fruits before and therefore know roughly how good or bad the experience of tasting a new fruit can possibly be. So, previous experiences of eating fruit (or food more generally) have led to the discovery of higher-order facts about eating fruit/food, which can then be applied to the experience of eating a durian. This allows for an approximate mental simulation of eating durian, from which we can then derive the possible subjective values of eating durian.^13^ In other words, we substitute the mental simulation of eating durian with a mental simulation of the higher-order properties that eating durian shares with experiences we are already familiar with. While this will not reveal the actual phenomenology of eating durian—leaving it epistemically transformative—it will inform us about the subjective values that eating durian can yield (cf. Villiger 2021).

Applied to the context of breaking up, rational agents should analyze whether previous experiences have led to the discovery of higher-order facts that also apply to the outcome of breaking up. If they did learn such higher-order facts, they can use them to run an approximate mental simulation of breaking up, which in some (but not all) cases will be reliable enough to make a rational decision. Such higher-order facts also seem to exist in our context. For example, having ended a previous relationship can give you some idea of what ending a next relationship will be like even though the two relationships are difficult to compare. Similarly, having been single before can give you some idea of what it will be like to be single again even if your singledom is many years ago and you have changed in the meantime. In each case, the relevant past experiences may enable you to run an approximate mental simulation of leaving your partner, thereby allowing you to put limits on the range of possible subjective values of that outcome. For instance, let’s assume that you have been left before and handled it fairly well, and that from earlier life stages you know you are the type of person who enjoys solitude. Based on these experiences, you might be able to run an approximate mental simulation of leaving your partner from which you can infer that its expected subjective value is unlikely to be highly negative and therefore unlikely to swamp the non-subjective value of leaving your partner if that value is strongly positive, making a rational choice possible.

Those seeking to avail themselves of this strategy face a complication, though: how can we know which, if any, of the higher-order facts accessible to us apply to a given transformative outcome? Reports from people who have already undergone the transformative experience can provide guidance here by helping to reveal which higher-order facts truly apply to the experience. For example, breaking up after a long relationship shares relevant higher-order properties (e.g., grief symptoms) with the loss of a loved one through death (cf. Petersen 2019). Given that we have already lost a loved one through death, we can partly build our approximate mental simulation of leaving our partner on that experience.

Nonetheless, even after informing ourselves about the relevant higher-order facts of breaking up, we are not always able to run an approximate mental simulation. First, breaking up may resemble experiences we have not yet encountered. Second, the information regarding which experiences are similar to breaking up may be inconclusive (cf. Paul 2014: 164). Third, there may be no experiences sufficiently similar to breaking up to enable an approximate mental simulation. Fourth, our past experience of relevant higher-order properties may no longer be reliable for how we would experience them today because we have personally changed significantly in the meantime. So, what to do when the strategy of simulating the outcome based on higher-order facts does not allow for a rational decision about whether to separate?

## Consider Subjective Values Derived by Other Agents (Level III)

Several authors have proposed testimony-based accounts of transformative decision-making that can deal with such cases (Dougherty et al. 2015; Pettigrew 2015; 2016b; 2019; Sharadin 2015). According to these accounts, what we should do is to see whether we can base our decision on the (average) subjective value derived by those who have already undergone the transformative experience. Thus, running a (approximate) mental simulation is replaced by exclusively consulting third-personal information.

The lack of mental simulation is the crucial difference between this strategy and the previous one: While in the previous strategy consulting third-personal information can also be important—namely, to gain knowledge about the higher-order properties of a transformative outcome—the purpose of doing so is to run a better approximate mental simulation. So, we still derive the possible subjective values through our own mental simulation and thus our own experience. This is no longer the case when we consider subjective values derived by other agents as a strategy for transformative decision-making. Here, we directly replace the unknown subjective value of a transformative experience with the values derived by those who have already undergone the transformative experience. For Paul, the fact that the subjective value is no longer derived from our own experience is a significant drawback of this strategy: we are no longer making the decision from a first-personal perspective because it is completely dependent on third-personal information. That is why this strategy is inferior to the one presented in the last section, which maintains a first-personal perspective, making the consideration of subjective values derived by other agents the third level in our five-level account.

For this strategy to work, it is best if the individuals whose subjective values we use share as many relationship factors (e.g., relationship duration, cohabitation, children, shared friends, etc.) and non-relationship factors (e.g., personality type, personal background, chronic illness, neurodivergence, etc.) with us as possible. The less overlap there is between us and those whose subjective values we use, ceteris paribus, the less likely those subjective values are to accurately represent our own subjective values. For instance, if someone broke up to escape their ex-partner’s abusive behavior, then insofar as our own partner is not abusive, we may not learn much from this person’s testimony (perhaps unless we share many non-relationship factors with them). Similarly, if someone is considerably more neurotic than we are, and has a very different personal background than we do, then that person’s breakup story may tell us little (perhaps unless we share many relationship factors with them). Since not everyone will know people who are comparable to them in terms of relationship and/or non-relationship factors, this significantly limits the usefulness of testimonies for deciding about a transformative breakup.

In addition to considering well-matched testimony, we can also turn to empirical studies and try to derive the subjective value of a transformative outcome from them. Typically, empirical studies have sample sizes that are much larger than what we could attain when collecting people’s testimony individually. However, the samples and subsamples tend to match us only in a very coarse-grained manner. This is why considering well-matched testimony is usually preferrable to considering empirical studies. But regardless of this limitation: What decision-relevant insights does the empirical literature on breakups offer?

The empirical literature on the effects of divorce on life satisfaction point to four moderators: initiation, severity of relationship problems, existence of children, and gender. First, if you are the initiator of divorce (or there is a shared initiation), your life satisfaction after divorce turns out higher compared to a unilaterally left partner (Symoens et al. 2013; Wang and Amato 2000). Second, if your marriage involves severe problems, divorce likely increases life satisfaction (at least in the case of women; in the case of men, it is less clear) (Amato and Hohmann-Marriott 2007; Bourassa, Sbarra, and Whisman 2015; Gustavson et al. 2012). In contrast, the effects of divorce on life satisfaction are rather negative in the case of low-distress marriages (Amato and Hohmann-Marriott 2007; Gustavson et al. 2012). Third, if you do not have children, divorce likely increases life satisfaction (Gardner and Oswald 2006). In contrast, if you do have children, divorce does not increase life satisfaction (but temporarily decreases it) if you are a man, or if you are a woman and your children are younger than five (Leopold and Kalmijn 2016).^14^

While these findings may allow us to derive the expected subjective value of breaking up in certain situations, they must be treated with caution: First, despite the identified moderators of the effects of divorce on life satisfaction, the overall picture presented by these studies remains very coarse. As a result, it is highly uncertain whether the subjective values derived from these findings truly reflect one’s own subjective values. Furthermore, the studies mainly focus on divorce among heterosexual couples, keeping us in ignorance of whether separation in non-marital and/or non-heteronormative relationships has similar effects (cf. Amato 2010). For example, there is evidence that homosexual couples have better post-dissolution relationships than heterosexual couples, which is likely to affect the impact of breaking up on life satisfaction (Lannutti and Cameron 2002). Second, while life satisfaction contributes to subjective value, it is not the only contributor.^15^ Third, empirical studies that rely on self-reporting can be biased because participants systematically misreport (cf. Jenkins 2016). In the context of breakups, a recent study suggests that participants report higher levels of happiness than they actually feel (Andreoni et al. 2024). Due to these limitations, we conclude that basing one’s decision to break up on empirical studies will not enable a rational choice unless the results are fairly clear-cut (cf. Paul 2015b).^16^

## Cut Down the Decision (Level IV)

What if neither moral values are decisive, nor can we derive the subjective value of the transformative outcome from an approximate mental simulation or directly from third-personal information? In such cases, we suggest that the logical next step for those seeking to decide about the fate of their relationship rationally will be to divide the decision into smaller decisions, if possible. In this way, the one big transformative decision may be turned into several smaller non-transformative sub-decisions. So, this strategy tries to circumvent the problems that transformative decisions pose to decision theory by changing the decision. Because of its indirect approach, cutting down the decision is inferior to the previous strategies for rational transformative choice, which directly resolve the decision at hand. This makes it the fourth level in our five-level account of transformative decision making.

We take our inspiration here from Ullmann-Margalit (2006), who proposes that, when possible, momentous life decisions should be broken up into two or more steps of which each is reversible and thus does not involve a huge leap. For example, regarding the transformative decision of whether getting married (cf. Paul 2014: 93–96), Ullmann-Margalit (2006) writes: “[I]f the big decision you face is whether to marry this man or not, you may try to arrange for the two of you to live together for a while so that you can get a foretaste of your future life—and of your future self—as his wife.” (169). In fact, this is what usually happens in a relationship anyway. You do not commit immediately, but the commitment is built up gradually: after going on the first dates, you might see each other more regularly, then you might get to know each other’s friends and family, then you might move in together, and so on. None of these steps might be transformative on their own even if their cumulation is by transforming you from a single into a person in a long-term, committed relationship. Carel and Kidd (2020) call such an experience a “cumulative transformative experience.” At this, the gradual building of a relationship likely reflects the experience of falling in love, which itself may often be a cumulative transformative one.

While the experience of falling out of love may also often be a cumulative transformative one, there is usually no gradual unraveling of the relationship reflecting this experience. Rather, breakups occur in a single moment, often during a conversation with one’s partner, and the consequences unfold immediately. Thus, unlike a relationship’s beginning, its end is usually not a cumulative transformative experience, which makes it harder to cut down the decision to end a relationship into non-transformative sub-decisions. One option for cutting down the breakup decision is to first have a relationship break for a fixed period of time, with you and your partner separating spatially if possible (Cherry 2022). Such a relationship break can give you a (better) sense of what it would be like to live without your partner and allows you to reflect on your partner and your relationship with some distance: for example, during the break you might realize how much you miss your partner and a clear desire to not give up on your relationship evolves; or you might realize that you appreciate the distance to your partner and that the relationship has no future.^17^

A relationship break as an intermediate step certainly makes sense when you are experiencing relationship ambivalence and are unsure whether to continue or end the relationship. However, it also comes with two major problems: First, while a break may help you gain clarity about whether to break up, for your partner, it is likely to be primarily stressful, unsettling, and destabilizing. And the more you distance yourself socially and emotionally from your partner to maximize the epistemic benefits of the break (e.g., by withdrawing completely from your partner and/or dating new people), the more negative the effects tend to be for your partner. This must be taken into account when defining the conditions of a relationship break. As a result, an ethical relationship break will not maximize its epistemic benefits at the expense of the partner’s well-being, making it less likely that the break will render the decision to end the relationship non-transformative.

Second, the result of the cutting down is still very coarse-grained, turning one decision (leaving yes/no) into two (relationship break yes/no; leaving yes/no). So, unlike the beginning of a relationship, you can only cut down the ending of a relationship into two sub-decisions, making it much less likely that both are non-transformative. The best way to minimize the decision-theoretic consequences of this problem is to make sure that the first sub-decision is non-transformative and thus unproblematic for decision theory. For example, you decide upon a relationship break that involves going to friend for a week or two. Such a break, which is mainly for deliberative purposes (but also gives you a tiny glimpse of what life is like without your partner), is hardly transformative and can be easily reversed.^18^ Still, it can be helpful, because during the reflective distance you may gain new insights that will allow you to make a rational decision about whether to leave your partner. And in the end, only through trial (and error) you get to know whether a relationship break renders the breakup-decision non-transformative.

## Consider the Revelatory Value (Level V)

The previous section suggested that cutting down the decision is not a magic bullet to make it non-transformative. So, there will be many cases where an agent has cut down the decision by having a relationship break first, but this has not made the next sub-decision—whether to break up—non-transformative. We argue that in such cases rational agents should follow Paul’s (2014) solution for rational transformative decision-making. In her view, the rational thing to do when one is confronted with a transformative decision is to consider the outcome’s revelatory value instead of its subjective value. Applied to the decision of whether leaving your partner, this approach says that you can rationally leave your partner if, and when, you want to find out how it is to live the outcome of leaving your partner *regardless* of how its subjective value turns out. If, however, you do not want to find out how it is to live the outcome of leaving your partner, you can rationally stay in the relationship.^19^ As can be seen, Paul’s revelatory approach allows you to make a kind of negative choice: If you are in a relationship break, know that you do not want to go back into that relationship, and thus want to find out what it is like to leave, you can rationally break up. Conversely, if the break does not resolve your ambivalence but you realize that you do not want to know what it is like to end this relationship, you can rationally stay with your partner. This provides a rational strategy for all breakup decisions that could not be solved by the previous four strategies of our five-level account.^20^

At this point, it must be noted that Paul’s revelatory approach has been criticized for its deus ex machina like characteristics (Bykvist and Stefánsson 2017; Callard 2018; Kauppinen 2015; Pettigrew 2019): Why should it suddenly be legitimate to base your decision on an outcome’s revelatory value and thereby be indifferent about its subjective value if the same is not legitimate in the case of non-subjective values (cf. Section 3)? While Paul does not provide an answer to this question, there is an important difference between the revelatory value and non-subjective values. The revelatory value is a second-order value of the subjective value. Thus, if we substitute the subjective value with the revelatory value, we do not completely neglect the subjective value but consider a second-order component of it. In contrast, if we base our decision on non-subjective values only, we completely ignore the subjective value. This provides a reason why, unlike in the case of non-subjective values, we can base a transformative decision on the revelatory value. Then again, if we use the revelatory value instead of the subjective value to make a decision, it is not guaranteed that the revelatory value will not get swamped by the unknown subjective value. Thus, choosing based on the revelatory value remains a leap of faith and amounts to what might be called “second-best rationality.” For this reason, it should only be used when other strategies that can control for potential swamping have failed, making it the fifth and final strategy in our five-level account.

## Conclusion

This is the first scholarly paper using rational decision theory to analyze one of the biggest and most momentous life decisions: whether to end one’s romantic relationship. Since ending a relationship often constitutes a transformative experience, particularly when one has been together for a long time and invested a lot in the relationship, the application of rational choice theory is not straightforward. Our analysis shows that even when a breakup is transformative, it is possible to reach a rational decision about it by following a novel five-level account of transformative decision-making, where one advances to the next level only if the previous one did not enable rational choice. First, one should consider whether moral values are decisive in telling one what to do. Second, one should try to restore rationality by running an approximate mental simulation of breaking up based on higher-order facts. Third, one should consider whether one can directly base one’s decision on subjective values derived by those who have already undergone the transformative experience. Fourth, one should try to render the decision to break up non-transformative by cutting it down. Fifth, one should base one’s decision on the revelatory value.

The upshot is that while decisions about transformative breakups are bound to remain difficult, there is reason for being optimistic that they can be made rationally. We hope that this contribution will spark more reflection on when it is rational to end romantic relationships, which we saw is a neglected question, as well as stimulate engagement with the five-level account of transformative decision-making developed here.

## Data Availability

Not applicable.

## Footnotes

1 Yet, in recent years, there has been a single positivity movement which fights against the stigma of being single and the amatonormative belief that being in a romantic relationship is the primary way to happiness (Cernik 2019).

2 Apart from the fact that it can, and frequently does, hurt to be rejected by those who until then played a prominent role in our lives, which may be accompanied by a loss of self-esteem (cf. Betzler 2023: 480), it is worth noting that unilaterally left parties not rarely still have feelings for their ex-partner. For a discussion of science-backed strategies to overcome these feelings, see Brogaard (2015: chap. 9). For a discussion of the potential moral duties of those who unilaterally ended a non-abusive committed relationship towards their ex-partners, see Betzler (2023).

3 Which, of course, is not to suggest that breaking up can never be a positive experience for one or both parties involved; for some people, it is. Additionally, Lopez-Cantero and Archer (2020) have highlighted that, insofar as breaking up is preceded or followed by the experience of falling out of love, this experience can be valuable, as it allows some of us to rediscover our own values and recognize how they might have been subsumed by those of one’s ex-partner.

4 We use the term transformative decision to refer to a decision that includes at least one option which, if chosen, has a non-negligible likelihood of leading to a transformative experience.

5 It is important to note that transformative experiences pose a challenge to decision theory only under a certain understanding of rational decision-making (for a detailed discussion, see Villiger 2024).

6 As noted in footnote 3, Lopez-Cantero and Archer (2020) believe that for people who sacrificed too much autonomy within a relationship, this process can potentially have value even if it is simultaneously stressful and disorienting.

7 Which is a situation in which women are particularly likely to find themselves (Andreb and Brockel 2007; Uunk, 2004). Another possible case where it might be rational to stay in a relationship, at least for the time being, irrespective of its subjective value is when one’s partner is violent and likely to physically assault one if one were to leave them. To deal with such cases, some places, including New York, have enacted mandatory arrest laws that require police officers to arrest individuals suspected of domestic violence when there is probable cause regardless of the victim’s consent (cf. Cook & Taylor, 2019; Iyengar, 2009).

8 Importantly, the decision whether to leave your partner also seems to be a first-personal choice; like the decision of whether having children or marrying your partner, it is a relationship decision concerning how and with whom you want to live your life.

9 Insofar as this type of normativity exists. For a skeptical take on prudential reasons, see Worsnip (2018).

10 As might be true if one is likely to be awarded sole custody of the children and if there is no realistic chance that the parent can be persuaded to cease their abusive conduct or that they will do so of their own accord. If, on the other hand, the abusive partner is likely to be awarded full or simply joint physical custody, the situation may not improve for the children; in fact, it could become worse when one is no longer around to restrain one’s partner.

11 Since the discussion of moral values is largely absent from the literature on transformative experiences, one might think that they are also of little importance in most transformative decisions, with breaking up being a major exception. But this is wrong. For example, moral values can also be important in the case of a career change or a move abroad: If you have a family to support, it may not be morally justifiable to leave your well-paying career and start a penniless career as an artist for the sake of self-fulfillment (as Paul Gaugin did, for example). Or if I have young children, it does not seem morally justifiable to leave them and move to another country for the sake of self-fulfillment (again as Paul Gaugin did).

12 To be clear, Kolodny (2003: 64-67) does believe that there can be sufficient reasons to fall out love with your romantic partner, in which case ending the romantic relationship becomes appropriate on his account. These reasons include that your partner no longer has concern for you; that they fail to act on the reasons supplied by your relationship; that their actions make them no longer worthy of respect; that you (correctly) no longer consider them the same person; and that you have ceased to be attracted to them, although some doubts are expressed about this last one (Kolodny 2003: 167).

13 To estimate the expected subjective value of eating durian, we need to know not only the range of possible subjective values but also how likely each value within that range is. This probability distribution can also be derived from higher-order knowledge (e.g., I have liked almost all the fruits I have tried so far, so the probability that I will also like this fruit is higher than the probability that I will not). If our higher-order knowledge does not allow us to form a specific probability distribution, we can, for example, apply the Principle of Indifference and use a uniform probability distribution (cf. Pettigrew 2016b, 2016a).

14 The fact that initiation, severity of relationship problems, and existence of children moderate the effect of divorce on life satisfaction seems intuitive. But what may explain the gender differences? We can think of two plausible explanations: (1) Women more often initiate divorce than men (Andreb and Brockel 2007). Since initiation of divorce correlates with better adjustment, we find overall better adjustment among women than men. (2) After divorce with children, men usually have much less contact with their children than women, resulting in a sharper decline in family well-being in the case of men which does not completely recover (Leopold and Kalmijn 2016). Due to that, life satisfaction after divorce is lower in the case of fathers compared to mothers.

15 Typically, emotional well-being is perceived to be strongly linked to happiness and, in that way, also to the subjective value, yet life satisfaction does not per se account for that connection (Haybron 2013).

16 In addition to these limitations, the cited studies remain silent on why breaking up affects life satisfaction in the way it does. One relevant factor seems to be social norms (cf. Jenkins 2016): being single is considered a deficient state in an amatonormative society, and being a divorcee is viewed as a life failure, among other examples. Study findings can reinforce these social norms if they are not critically examined. Therefore, agents who consult empirical studies (and/or individual testimony) for their decision-making should reflect on the social norms that may underlie participants’ responses, and consider how participants might have answered in the absence of these norms.

17 Many countries seem to encourage such breaks by having legally mandated waiting periods for couples seeking a divorce, which have been found to decrease the number of finalized divorces especially for people who were in a fragile emotional state when they initiated the divorce proceedings (Lee 2013; Wie and Kim 2015).

18 In contrast, a longer relationship break that involves moving out, and possibly even dating new people (to assess what it would be like to be with another partner), is likely to be transformative. This is both because the experience itself can be transformative and because it carries the risk that your partner may not want you back, resulting in an (unwanted) breakup.

19 Here, we assume that the assessable (non-subjective) value of continuing and ending the relationship are more or less equal, which is why the revelatory value becomes decisive. Thus, non-subjective values are not irrelevant in the revelatory approach and able to swamp a positive or negative revelatory value. But this is not a problem because we know whether they do swamp the revelatory value and can choose accordingly.

20 If an agent is ambivalent about whether they want to find out what the outcome of breaking up is like, with the result that breaking up has neither positive nor negative revelatory value, they can rationally pick between continuing and ending the relationship.

## References

[CR1] Amato, Paul R. 2010. Research on divorce: continuing trends and new developments. *Journal of Marriage and Family* 72(3): 650–666.

[CR2] Amato, Paul R., and Bryndl Hohmann-Marriott. 2007. A comparison of high- and low-distress marriages that end in divorce. *Journal of Marriage and Family* 69(3): 621–638. 10.1111/j.1741-3737.2007.00396.x.

[CR3] Andreoni, James, B. Douglas Bernheim, and Tingyan Jia. 2024. ‘Do People Report Happiness Accurately?’ National Bureau of Economic Research. https://www.nber.org/papers/w32208.

[CR4] Andreß, H.J., and M. Bröckel. 2007. Income and life satisfaction after marital disruption in Germany. *Journal of Marriage and Family* 69(2): 500–512. 10.1111/j.1741-3737.2007.00379.x.

[CR5] Austin, M.W. 2016. *Conceptions of parenthood: ethics and the family*. Farnham: Ashgate Publishing.

[CR6] Barnes, E. 2015. What you can expect when you don’t want to be expecting. *Philosophy and Phenomenological Research* 91(3): 775–786.

[CR7] Betzler, M. 2023. The right to associational freedom and the scope of relationship-dependent duties. *Criminal Law and Philosophy* 17(2): 475–489. 10.1007/s11572-022-09640-w.

[CR8] Blow, A.J., and K. Hartnett. 2005. Infidelity in committed relationships I: a substantive review. *Journal of Marital and Family Therapy* 31(2): 217–233. 10.1111/j.1752-0606.2005.tb01556.x. 15974059 10.1111/j.1752-0606.2005.tb01556.x

[CR9] Blustein, J. 1982. *Parents and children: the ethics of the family*. Oxford: Oxford University Press.

[CR10] Bourassa, K.J., D.A. Sbarra, and M.A. Whisman. 2015. Women in very low quality marriages gain life satisfaction following divorce. *Journal of Family Psychology* 29(3): 490. 25868007 10.1037/fam0000075 PMC12063491

[CR11] Brake, E. 2011. Is divorce promise-breaking? *Ethical Theory and Moral Practice* 14(1): 23–39.

[CR12] Brighouse, H., and A. Swift. 2014. *Family values: the ethics of parent-child relationships*. Princeton: Princeton University Press.

[CR13] Brogaard, Berit. 2015. *On romantic love: simple truths about a complex emotion*, 1st ed. New York: Oxford University Press.

[CR14] Brown, Anna. 2020. ‘Nearly Half of U.S. Adults Say Dating Has Gotten Harder for Most People in the Last 10 Years’. *Pew Research Center’s Social & Demographic Trends Project* (blog). 20 August 2020. https://www.pewresearch.org/social-trends/2020/08/20/nearly-half-of-u-s-adults-say-dating-has-gotten-harder-for-most-people-in-the-last-10-years/.

[CR15] Buss, D.M. 2018. Sexual and emotional infidelity: evolved gender differences in jealousy prove robust and replicable. *Perspectives on Psychological Science* 13(2): 155–160. 10.1177/1745691617698225. 29592639 10.1177/1745691617698225

[CR16] Bykvist, K., and H. OrriStefánsson. 2017. Epistemic transformation and rational choice. *Economics and Philosophy* 33(1): 125–138.

[CR17] Callard, Agnes. 2018. *Aspiration: the agency of becoming*. New York: Oxford University Press.

[CR18] Carel, H., and I.J. Kidd. 2020. Expanding transformative experience. *European Journal of Philosophy* 28(1): 199–213. 32421021 10.1111/ejop.12480 PMC7217260

[CR19] Cath, Yuri. 2019. Knowing what it is like and testimony. *Australasian Journal of Philosophy* 97(1): 105–120.

[CR20] Cernik, Lizzie. 2019. ‘Self-Partnered: The Sudden, Surprising Rise of the Single Positivity Movement’. *The Guardian*, 6 November 2019, sec. Life and style. https://www.theguardian.com/lifeandstyle/2019/nov/06/consciously-uncoupled-the-joy-of-self-partnership.

[CR21] Cherry, Kendra. 2022. ‘Do Breaks In Relationships Work?’ Verywell Mind. 13 October 2022. https://www.verywellmind.com/do-breaks-in-relationships-work-5219589.

[CR22] Chiesa, A.E., L. Kallechey, C. Nicole Harlaar, R. Ford, E.F. Garrido, W.R. Betts, and S. Maguire. 2018. Intimate partner violence victimization and parenting: a systematic review. *Child Abuse & Neglect* 80 (June): 285–300. 10.1016/j.chiabu.2018.03.028. 29665506 10.1016/j.chiabu.2018.03.028

[CR23] Cook, J.A., and T.W. Taylor. 2019. The impact of mandatory arrest laws on domestic violence in times of economic stress. *Economics Letters* 178 (May): 77–81. 10.1016/j.econlet.2019.02.013.

[CR24] Copp, D. 2015. Rationality and moral authority. *Oxford Studies in Metaethics*. 10.1093/acprof:oso/9780198738695.003.0006.

[CR25] Cowley, C. 2020. Divorce, disorientation, and remarriage. *Ethical Theory and Moral Practice* 23(3): 531–544. 10.1007/s10677-019-10036-4.

[CR26] Cowley, Christopher. 2021. “Relational Vertigo” in before midnight. In *Before Sunrise, Before Sunset, Before Midnight*. Abingdon: Routledge.

[CR27] Dougherty, T., S. Horowitz, and P. Sliwa. 2015. Expecting the unexpected. *Res Philosophica* 92(2): 301–321.

[CR28] Edleson, J. 1999. Children’s witnessing of adult domestic violence. *Journal of Interpersonal Violence* 14 (8): 839–870. 10.1177/088626099014008004.

[CR29] Garbinsky, E.N., J.J. Gladstone, H. Nikolova, and J.G. Olson. 2020. Love, lies, and money: financial infidelity in romantic relationships. *Journal of Consumer Research* 47(1): 1–24.

[CR30] Gardner, J., and A.J. Oswald. 2006. Do divorcing couples become happier by breaking up? *Journal of the Royal Statistical Society: Series A (Statistics in Society)* 169(2): 319–336. 10.1111/j.1467-985X.2006.00403.x.

[CR31] Gauthier, David. 1986. *Morals by agreement*. Oxford: Clarendon Press.

[CR32] Ge, F., J. Lembke, and P.R. Pietromonaco. 2020. Intimate relationships and physical health. In *The Wiley Encyclopedia of Health Psychology*, 337–345. Hoboken: John Wiley & Sons, Ltd.

[CR33] Gheaus, Anca. 2018. ‘Personal Relationship Goods’. In *The Stanford Encyclopedia of Philosophy*, ed. Edward N. Zalta, Fall 2018. Metaphysics Research Lab, Stanford University. https://plato.stanford.edu/archives/fall2018/entries/personal-relationship-goods/.

[CR34] Gustavson, K., E. Røysamb, T. von Soest, M.J. Helland, and K.S. Mathiesen. 2012. Longitudinal associations between relationship problems, divorce, and life satisfaction: findings from a 15-year population-based study. *The Journal of Positive Psychology* 7(3): 188–197.

[CR35] Härkönen, J., F. Bernardi, and D. Boertien. 2017. Family dynamics and child outcomes: an overview of research and open questions. *European Journal of Population* 33(2): 163–184. 10.1007/s10680-017-9424-6. 30976231 10.1007/s10680-017-9424-6 PMC6240988

[CR36] Haybron, D.M. 2013. *Happiness - a very short introduction*. Oxford: Oxford University Press.

[CR37] Helm, Bennett. 2021. ‘Love’. In *The Stanford Encyclopedia of Philosophy*, ed. Edward N. Zalta, Fall 2021. Metaphysics Research Lab, Stanford University. https://plato.stanford.edu/archives/fall2021/entries/love/.

[CR38] Hobson, C.J., L. Delunas, and D. Kesic. 2001. Compelling evidence of the need for corporate work/life balance initiatives: results from a national survey of stressful life-events. *Journal of Employment Counseling* 38(1): 38–44. 10.1002/j.2161-1920.2001.tb00491.x.

[CR39] Iyengar, R. 2009. Does the certainty of arrest reduce domestic violence? Evidence from mandatory and recommended arrest laws. *Journal of Public Economics* 93(1): 85–98. 10.1016/j.jpubeco.2008.09.006.

[CR40] Jenkins, C.S.I. 2016. Knowing our own hearts: self-reporting and the science of love. *Philosophical Issues* 26: 226–242.

[CR41] Johnsona, R.M., J.B. Kotch, D.J. Catellier, J.R. Winsor, V. Dufort, W. Hunter, and L. Amaya-Jackson. 2002. Adverse behavioral and emotional outcomes from child abuse and witnessed violence. *Child Maltreatment* 7(3): 179–186. 10.1177/1077559502007003001. 12139186 10.1177/1077559502007003001

[CR42] Jollimore, Troy. 2017. Love as “Something in Between.” In *The Oxford Handbook of Philosophy of Love*, ed. Christopher Grau and Aaron Smuts. Oxford: Oxford University Press.

[CR43] Kant, I. 1996. The metaphysics of morals. In *Gregor*, ed. J. Mary. Cambridge: Cambridge University Press.

[CR44] Kauppinen, A. 2015. What’s so great about experience? *Res Philosophica* 92(2): 371–388.

[CR45] Kind, Amy. 2020. What imagination teaches. In *Becoming Someone New: Essays on Transformative Experience, Choice, and Change*, ed. Enoch Lambert and John Schwenkler, 133–146. Oxford: Oxford University Press.

[CR46] Kolodny, N. 2003. Love as valuing a relationship. *Philosophical Review* 112(2): 135–189. 10.1215/00318108-112-2-135.

[CR47] Korsgaard, C.M. 2009. *Self-constitution: agency, identity, and integrity*, 1st ed. Oxford: Oxford University Press.

[CR48] Lannutti, P.J., and K.A. Cameron. 2002. Beyond the breakup: heterosexual and homosexual post-dissolutional relationships. *Communication Quarterly* 50(2): 153–170. 10.1080/01463370209385654.

[CR49] Lee, J. 2013. The impact of a mandatory cooling-off period on divorce. *The Journal of Law and Economics* 56(1): 227–243. 10.1086/667710.

[CR50] Leopold, T., and M. Kalmijn. 2016. Is divorce more painful when couples have children? Evidence from long-term panel data on multiple domains of well-being. *Demography* 53(6): 1717–1742. 27815739 10.1007/s13524-016-0518-2 PMC5127958

[CR51] Liberman, A. 2021. For better or for worse: when are uncertain wedding vows permissible? *Social Theory & Practice* 47(4): 765–788. 10.5840/soctheorpract2021923141.

[CR52] Locker, L., W.D. McIntosh, A.A. Hackney, J.H. Wilson, and K.E. Wiegand. 2010. The breakup of romantic relationships: situational predictors of perception of recovery. *North American Journal of Psychology* 12(3): 1.

[CR53] Lopez-Cantero, P. 2018. The break-up check: exploring romantic love through relationship terminations. *Philosophia* 46(3): 689–703. 10.1007/s11406-017-9935-8. 30147189 10.1007/s11406-017-9935-8 PMC6096559

[CR54] Lopez-Cantero, P., and A. Archer. 2020. Lost without you: the value of falling out of love. *Ethical Theory and Moral Practice* 23(3): 515–529. 10.1007/s10677-020-10067-2.

[CR55] McKeever, N., and J. Saunders. 2022. Irrational love: taking Romeo and Juliet seriously. *International Journal of Philosophical Studies* 30(3): 254–275. 10.1080/09672559.2022.2121895.

[CR56] Muñoz, Daniel. 2022. ‘Obligations to Oneself’. In *The Stanford Encyclopedia of Philosophy*, ed. Edward N. Zalta, Summer 2022. Metaphysics Research Lab, Stanford University. https://plato.stanford.edu/archives/sum2022/entries/self-obligations/.

[CR57] Naar, H. 2022. *The rationality of love*. Oxford: Oxford University Press.

[CR58] O’Neill, O. 1979. *Having children: philosophical and legal reflections on parenthood*. Oxford: Oxford University Press.

[CR59] Paul, L.A. 2014. *Transformative experience*, 1st ed. Oxford: Oxford University Press.

[CR60] Paul, L.A. 2015. Transformative choice: discussion and replies. *Res Philosophica* 92(2): 473–545.

[CR61] Paul, L.A. 2015. Transformative experience: replies to Pettigrew, Barnes and Campbell. *Philosophy and Phenomenological Research* 91(3): 794–813. 10.1111/phpr.12250.

[CR62] Paul, L.A. 2015. What you can’t expect when you’re expecting. *Res Philosophica* 92(2): 149–170.

[CR63] Petersen, K. 2019. Breaking up Is Hard to Do. *Irish Association for Counselling and Psychotherapy* 19(3): 15–22.

[CR64] Pettigrew, R. 2015. Transformative experience and decision theory. *Philosophy and Phenomenological Research* 91(3): 766–774. 10.1111/phpr.12240.

[CR65] Pettigrew, R. 2016. Accuracy, risk, and the principle of indifference. *Philosophy and Phenomenological Research* 92(1): 35–59. 10.1111/phpr.12097.

[CR66] Pettigrew, R. 2016. Transformative experience, by L. A. Paul. *Mind* 125(499): 927–935. 10.1093/mind/fzw014.

[CR67] Pettigrew, R. 2019. *Choosing for changing selves*, 1st ed. Oxford: Oxford University Press.

[CR68] Reuter, K., and M. Messerli. 2018. Transformative decisions. *The Journal of Philosophy* 115(6): 313–335. 10.5840/jphil2018115620.

[CR69] Rosenfeld, M.J. 2014. Couple longevity in the era of same-sex marriage in the United States. *Journal of Marriage and Family* 76(5): 905–918. 10.1111/jomf.12141.

[CR70] Saunders, Joe. 2022. Loves passed. In *Philosophy of Love in the Past, Present, and Future*. Abingdon: Routledge.

[CR71] Sharadin, N. 2015. How you can reasonably form expectations when you’re expecting. *Res Philosophica* 92(2): 441–452.

[CR72] Sprecher, S., D. Felmlee, S. Metts, B. Fehr, and D. Vanni. 1998. Factors associated with distress following the breakup of a close relationship. *Journal of Social and Personal Relationships* 15(6): 791–809. 10.1177/0265407598156005.

[CR73] Steiner, L.M., E.C. Suarez, J.N. Sells, and S.D. Wykes. 2011. Effect of age, initiator status, and infidelity on women’s divorce adjustment. *Journal of Divorce & Remarriage* 52(1): 33–47.

[CR74] Steiner, L.M., S. Durand, D. Groves, and C. Rozzell. 2015. Effect of infidelity, initiator status, and spiritual well-being on men’s divorce adjustment. *Journal of Divorce & Remarriage* 56(2): 95–108.

[CR75] Symoens, S., K. Bastaits, D. Mortelmans, and P. Bracke. 2013. Breaking up, breaking hearts? Characteristics of the divorce process and well-being after divorce. *Journal of Divorce & Remarriage* 54(3): 177–196. 10.1080/10502556.2013.773792.

[CR76] Trachsel, Manuel, Lara Ferrari, and Martin Grosse Holtforth. 2012. ‘Resolving Partnership Ambivalence: A Randomized Controlled Trial of Very Brief Cognitive and Experiential Interventions with Follow-Up’. *Canadian Journal of Counselling and Psychotherapy* 46(3). https://cjc-rcc.ucalgary.ca/article/view/59855.

[CR77] Ullmann-Margalit, E. 2006. Big decisions: opting, converting, drifting. *Royal Institute of Philosophy Supplements* 81(58): 157–172.

[CR78] Uunk, W. 2004. The economic consequences of divorce for women in the European Union: the impact of welfare state arrangements. *European Journal of Population / Revue Européenne de Démographie* 20(3): 251–285.

[CR79] Villiger, D. 2021. A rational route to transformative decisions. *Synthese* 199(5): 14535–14553. 10.1007/s11229-021-03432-w. 35058667 10.1007/s11229-021-03432-w PMC8727407

[CR80] Villiger, D. 2022. The role of expectations in transformative experiences. *Philosophical Psychology*. 10.1080/09515089.2022.2070063.

[CR81] Villiger, D. 2023. Rational transformative decision-making. *Synthese* 201(3): 87. 10.1007/s11229-023-04075-9. 36855375 10.1007/s11229-023-04075-9 PMC9958159

[CR82] Villiger, D. 2024. Transformative experience. *Philosophy Compass* 19(6): e13000. 10.1111/phc3.13000.

[CR83] Wang, H., and P.R. Amato. 2000. Predictors of divorce adjustment: stressors, resources, and definitions. *Journal of Marriage and Family* 62(3): 655–668. 10.1111/j.1741-3737.2000.00655.x.

[CR84] Wie, D., and H. Kim. 2015. Between calm and passion: the cooling-off period and divorce decisions in Korea. *Feminist Economics* 21(2): 187–214. 10.1080/13545701.2014.999008.

[CR85] Worsnip, Alex. 2018. Eliminating prudential reasons. In *Oxford Studies in Normative Ethics*, ed. Mark C. Timmons. Oxford: Oxford University Press.

[CR86] Zayas, V., G. Surenkok, and G. Pandey. 2017. Implicit ambivalence of significant others: significant others trigger positive and negative evaluations. *Social and Personality Psychology Compass* 11(11): e12360. 10.1111/spc3.12360.

[CR87] Zoppolat, G., F. Righetti, R. Faure, and I.K. Schneider. 2024. A systematic study of ambivalence and well-being in romantic relationships. *Social Psychological and Personality Science* 15(3): 329–339. 10.1177/19485506231165585.

